# Hematological parameters and associated factors among adult patients with type 2 diabetes mellitus attending selected hospitals in Garowe, Puntland, Somalia: A comparative cross-sectional study

**DOI:** 10.1371/journal.pone.0353173

**Published:** 2026-07-16

**Authors:** Mohamud Abshir Musse, Haftu Asmerom, Mesay Arkew, Rajesh Sarkar, Winner Kucha

**Affiliations:** 1 Department of Medical Laboratory Sciences, Faculty of Medicine and Health Sciences, University of Bosaso, Garowe, Somalia; 2 School of Medical Laboratory Science, College of Health and Medical Science, Haramaya University, Ethiopia; King Faisal University, SAUDI ARABIA

## Abstract

**Background:**

Diabetes mellitus is characterized by elevated blood glucose levels resulting from inadequate insulin production or ineffective insulin utilization. Type 2 diabetes, the most prevalent form, is associated with alterations in hematological parameters. Notably, there is no evidence addressing hematological parameters and their associated factors among adult patients with type 2 diabetes mellitus in Garowe City. Thus, this study aimed to assess hematological parameters and associated factors among adult patients with type 2 diabetes mellitus in comparison to healthy controls attending hospitals in Garowe, Puntland, Somalia, from October 1 to November 15, 2024.

**Method:**

A comparative cross-sectional study was conducted 266 participants (133 type 2 diabetic patients and 133 comparative groups) using a systematic random sampling technique. Data were collected using questionnaires, checklists, and laboratory tests. Hematological parameters, fasting blood glucose, and glycated hemoglobin were measured. The collected data were entered into Epi-data v3.1, and exported to STATA v17 for analysis. Independent t-test, Mann-Whitney U-test, and correlation were used. A multivariable logistic regression model was used to see associations between predictors and outcomes. A *p*-value < 0.05 was considered statistically significant.

**Results:**

The current study found that the absolute counts of neutrophil, mean corpuscular volume, mean corpuscular hemoglobin concentration, mean platelet volume, and platelet distribution width were significantly different between type 2 diabetic patients and the comparative group (*p* < 0.05). Furthermore, the most prevalent hematological abnormalities among type 2 diabetic patients were anemia (31.6%), monocytosis (23.3%), thrombocytopenia (7.5%), and leukocytosis (6.8%). Multivariable analysis indicated that poor glycemic control was significantly associated with anemia (AOR = 3.06, 95% CI: 1.06–8.8), while systolic blood pressure > 140 mmHg was significantly associated with leukocytosis (AOR = 6.09, 95% CI: 1.09–33.9) in type 2 diabetic patients.

**Conclusions:**

Absolute neutrophil count, mean corpuscular volume, mean corpuscular hemoglobin concentration, mean platelet volume, and platelet distribution width differed significantly between type 2 diabetes mellitus patients and the comparative group. Anemia, monocytosis, thrombocytopenia, and leukocytosis were the most frequently observed hematological abnormalities in both groups. Poor glycemic control was significantly associated with anemia, while elevated systolic blood pressure was associated with leukocytosis among type 2 diabetes mellitus patients. The occurrence of anemia in both groups suggests that factors beyond diabetes may contribute to its burden in this population.

## Introduction

Diabetes mellitus (DM) is a long-term metabolic condition that leads to high blood sugar levels related to either problems with insulin production, insulin function, or both [1]. The global prevalence of diabetes has increased substantially, from about 200 million individuals in 1990 to nearly 830 million in 2022, with low- and middle-income countries experiencing the fastest rate of increase [2]. The prevalence of diabetes in Somalia has increased, rising from 5% in 2016 [3] to 6.5% in 2021 [4]

Chronic hyperglycemia is responsible for various physiological issues, including vasodilation, inflammatory regulation, and hematological parameters [5]. Several factors, such as elevated reactive oxygen species production, and the formation of advanced glycation end products (AGEs) due to chronic hyperglycemia, might contribute to hematological alterations in diabetes. Increased reactive oxygen species production leads to oxidative stress, which is linked to hematological changes like endothelium dysfunction, platelet (PLT) hyperactivity, and red blood cell (RBC) dysfunction as well as tissue damage [6].

Several hematological parameters, including RBC, white blood cell (WBC), and PLT counts, are associated with type 2 diabetes mellitus (T2DM) [7]. In patients with diabetes, RBCs undergo significant alterations in both morphological structure and physiological functions due to glucose metabolism disorders [8]. These alterations can impair blood flow, cause hypoxia, and increase OS, which can lead to anemia [9]. Glycated hemoglobin (HbA1c) increases the formation of highly reactive free radicals inside the RBCs, thus altering their cell membrane properties, leading to blood cell aggregation and increased blood viscosity, with a concomitant impaired blood flow in severe cases like DM [10].

Another hematological parameter change has been observed in WBC count that is associated with insulin resistance (IR) and the development of T2DM [11]. The chronic inflammation in diabetes causes leukocytosis, contributing to diabetic macro- and microvascular complications [12]. Platelet count, increased mean platelet volume (MPV), platelet distribution width (PDW), and plateletcrit (PCT) have been linked to endothelial dysfunction and inflammation-related conditions like metabolic syndrome, and diabetes [13]. Platelet hyperactivity and altered morphology in T2DM promote clot formation and thrombotic complications [14,15].

Hematological abnormalities such as anemia, leukocytosis, and thrombocytosis are frequently reported in T2DM patients. The prevalence of anemia ranges globally from 8.06% to 67% [16,17], contributing to complications such as neuropathy, nephropathy, retinopathy and increased cardiovascular risk [18]. Poor glycemic control and renal impairment further increase the likelihood of anemia in diabetic patients [19]. According to a systematic review and meta-analysis of cross-sectional and prospective studies, patients with T2DM had an increase in peripheral WBCs such as neutrophils, eosinophils, and basophils but no change in monocyte counts [20]. Moreover, platelet dysfunction in T2DM plays a central role in thromboembolic events, such as acute coronary syndromes [21].

Notably, there is no evidence addressing hematological parameters, and their associated factors among adult patients with T2DM in Garowe City. This research gap highlights those hematological parameters are not routinely used as diagnostic biomarkers to monitor diabetes, and its associated complications. Therefore, this study aimed to assess hematological parameters and associated factors among adult patients with T2DM in comparison to healthy controls attending selected hospitals in Garowe, Puntland, Somalia.

## Materials and methods

### Study design, area and period

A hospital-based comparative cross-sectional study was conducted from October 1 to November 15, 2024, at Garowe General Hospital, Arafat International Hospital, and Dalab Hospital in Garowe, Puntland, Somalia. Garowe, the capital and administrative center of Puntland State, is located in the Nugaal region of northeastern Somalia. Since the establishment of Puntland in 1998, the city has undergone significant urban growth, emerging as a major political and economic hub. The selected hospitals provide comprehensive healthcare services, including emergency care, chronic disease management, antiretroviral therapy, surgery, pediatrics, gynecology, obstetrics, and dental care, and more, for both inpatient and outpatient populations.

### Study participants

All adult T2DM patients who visited the selected hospitals in Garowe, Puntland, Somalia was included as the diabetic group. In addition, age and sex matched healthy individuals who attended the hospitals as blood donors or for medical checkups during the study period were involved as the comparative group. Controls were recruited using a 1:1 individual matching approach, whereby each T2DM patient was matched with one apparently healthy control by sex and age (±2 years). However, both T2DM patients and healthy individuals with chronic diseases (such as renal failure and liver disease), a history of infectious diseases including HIV/AIDS, HBV, and HCV, those taking hormone therapy, anticoagulant therapy, erythropoietin, or antiplatelet drugs, pregnant women, alcoholics, and individuals younger than 18 years or older than 65 years were excluded. Participants were also excluded if they met any exclusion criteria after reviewing medical records and face-to-face interviews.

### Sample size determination and sampling technique

The sample size was determined using a double population mean formula with Open-Epi statistical free software version 3.0.1, by considering the following assumptions: 95% confidence level, (2-tailed, α = 0.05), 80% power (β = 0.20), and the ratio of sample size (T2DM/comparative groups) was 1:1. The mean and standard deviation (SD) [mean ± SD] of Lymphocyte (10^3^/μl), for T2DM and comparative groups were taken from a study conducted in Debre Berhan, Ethiopia [22], which reported 2.07 ± 0.62 for T2DM, and 1.86 ± 0.54 for the control group.

After adding a 10% non-respondent rate, the sample size was calculated to be 133 for each group, and a total of 266 study participants were included in this study. A systematic random sampling technique was used.

### Data collection and laboratory analysis

Data related to socio-demographic, and lifestyle characteristics were collected using a pre-tested structured questionnaire through face-to-face interviews. Clinical variables, including duration of diabetes, duration of oral hypoglycemic therapy, as well as the type of therapy used, were obtained from the diabetic patients’ medical records using a checklist. After the participants rested for at least 5 minutes, blood pressure (BP) measurements were taken by clinical nurses from the upper part of the left arm at heart level using an analog sphygmomanometer and stethoscope.

On other hands, anthropometric variables such as height (to the nearest 0.1 centimeters) without shoes were measured using a stadiometer scale, and weight (to the nearest 0.1 kg) with the participant wearing very light clothing and no shoes was used the weighing scale. Body mass index (BMI) was calculated as weight in kilograms (kg) divided by height in meters squared, and they were classified as underweight (< 18.5 kg/m^2^), normal (18.5–24.9 kg/m^2^), overweight (25–29.9 kg/m^2^), and obese (≥ 30 kg/m^2^) [23]. Waist circumference (WC) was measured with a tape at the midway between the least palpable inferior margin of the rib and the iliac crest in the participant’s normal exhaled state. The hip circumference (HC) was also measured using the same tape around the widest portion of the buttocks, and the waist-to-hip ratio (WHR) was computed by dividing WC in centimeters by HC, collected according to the anthropometric measurements protocol. After an interview, review of medical records, anthropometric, and BP measurement were completed by trained clinical nurses, the study participants were sent to a laboratory where a blood sample was collected for determination of fasting blood glucose (FBG), HbA1c and hematological parameters.

Five milliliters (ml) of venous blood [2 ml in serum separator tube (SST) and 3 ml in Dipotassium Ethylenediaminetetraacetic Acid (K_2_-EDTA) tube] were collected from each of the T2DM patients and comparative groups (blood donors and medical checkups) by laboratory professionals after overnight fasting. Serum prepared from the SST was used to determine FBG. Fasting blood glucose concentrations were estimated using the glucose oxidase method, utilizing the Mindray BA-88A Semi-Auto Clinical Chemistry Analyzer (Mindray Bio-Medical Electronics Co., Ltd. in Shenzhen, China). The whole blood sample in the K_2_-EDTA tube was used to determine hematological parameters, and HbA1c. Hematological parameters were analyzed using a complete blood count (CBC) with the Zybio Semi-Automatic Hematology Analyzer Z5 (Dadukou District, Chongqing Municipality, China). Additionally, HbA1c levels in all T2DM patients were determined using the semi-automated I-CHROMA^TM^ II (Boditech Med Inc., Gangwon-do, Republic of Korea). Fasting blood glucose, HbA1C, and hematological parameters [RBC, Hgb, Hct, mean corpuscular volume (MCV), mean corpuscular hemoglobin (MCH), mean corpuscular hemoglobin concentration (MCHC), red cell distribution width–standard deviation (RDW-SD), red cell distribution width–coefficient of variation (RDW-CV), WBCs, absolute neutrophils, monocyte, lymphocytes, basophil, eosinophil, platelet count, MPV, PDW, and plateletcrit (PCT)] were analyzed at Arafat International Hospital laboratory and recorded for each study participants using laboratory result registration form.

### Operational definitions

**Hematological abnormalities:** Having any hematological alterations including anemia, thrombocytopenia, thrombocytosis, leukocytosis

**Hematological parameters:** The various measures of blood components that are evaluated in a complete blood count tests (i.e., RBC, Hgb, Hct, MCV, MCH, MCHC, RDW-SD, RDW-CV, WBC, WBC differential count, PLT, MPV, PDW and PCT).

**Anemia:** is defined as Hgb levels of < 12.0 g/dl for women, and < 13.0 g/dl for men; however, smoking and other factors also affect normal Hgb levels in addition to sex. Adjustments based on Hgb were calculated as fallows: smokers (g/L) = (0.4565 x cigarette number) + (− 0.0078 x cigarette_number^2^) [2].

**Glycemic control:** A HbA1c value of < 7% is considered good glycemic control, as recommended by the American Diabetes Association for non-pregnant adults, while a HbA1c value of ≥ 7% is considered poor glycemic control [24].

### Data quality assurance and management

The questionnaires and informed consent forms written in English were translated into the local language (Af-Somali), and retranslated into English for accuracy, and consistency. Before the actual data collection, questionnaires were pre-tested, and two-day training was given to data collectors. Anthropometric and BP measurements were taken three times, and the average value was used for data analysis.

The blood sample were collected and handled following the standard operating procedures (SOP) for specimen collection, CBC analysis, HbA1c, and FBG measurement to ensure the quality of laboratory data. Additionally, daily background checks were conducted routinely. Automated analyzers and other equipment were cleaned daily as a routine activity before closing the laboratory. All laboratory assays were conducted within 2 hours of sample collection. A blood film examination was performed for suspected flags in the analyzer. The completeness, and clarity of the collected data were checked regularly and the results were properly recorded, transcribed, and reviewed.

### Data processing and analysis

The data was checked for completeness and internal consistency, data was coded, entered, and cleaned using Epi Data version 3.1 (Epi Data Association, Odense, Denmark) computer software packages and exported to STATA version 17 software (Stata Corporation, Texas, USA) for analysis. To assess the normality of the data distribution, histograms, and Shapiro-Wilk test were utilized. Categorical variable results were presented as frequencies, and percentages. Statistical differences in these categorical variables were checked using the chi-square test. Normally distributed continuous variables were summarized using the mean ± SD, whereas skewed continuous variables were described using the median and IQR. The comparison of hematological parameters between T2DM patients and comparative group participants was done by independent t-test for normally distributed data, and Mann-Whitney U test for non-normally distributed data. Pearson’s correlation was used for normally distributed data, while Spearman’s correlation was used for non-normally distributed data to assess the correlation between hematological parameters and independent variables. A bivariable and Multivariable logistic regression analysis was used to assess the associations of predictors and outcomes with crude odds ratios (COR), and adjusted odds ratios (AOR) with 95% confidence intervals (CI). Variables with p-values < 0.25 were included for multivariable logistic regression analysis. The results were summarized, and presented using tables and texts. Finally, P-value of < 0.05 was declared as statistical significance.

### Ethical consideration

Ethical clearance was obtained from the Institutional Health Research Ethics Review Committee (IHRERC) of Haramaya University, College of Health and Medical Sciences with letter reference number IHRERC/240/2024. A letter of permission was obtained from the district administrative health bureau. Before starting sample collection, permission was obtained from each participant using written consent. Information on the study was explained to each participant including objectives, procedure, potential risks, and benefits of the study. The study participants were informed of their right to refuse or withdraw from the study at any time. Refusing to participate in the study did not affect each study participant. Then informed, voluntary, written, and signed consent was taken from each head of hospital, and participant. To protect the privacy of the study participants, they were assigned unique codes instead of using their identifiers.

## Results

### Socio-demographic characteristics

This study included a total of 266 participants, with 133 in the T2DM patients, and 133 in the comparative group. The median age of participants was 50 years (IQR: 40–56) among patients with T2DM patients and 49 years (IQR: 39–55) in the comparative group. While, 124 (93.23%) of the participants in the T2DM patients and 121 (90.98%) in the comparative group resided in urban areas. Regarding education level, 55 (41.35%) of the T2DM patients, and 43 (32.33%) of the comparative group could read and write. Additionally, 45 (33.83%) of the T2DM patients, and 26 (19.55%) of the comparative group were housewives Table 1.

**Table 1:** Socio-demographic characteristics of adult patients with type 2 diabetes mellitus and a comparative group at selected hospitals in Garowe, Puntland, Somalia, from October 1 to November 15, 2024 (n=266).

Variables	Categories	T2DM Patients (n = 133)	Comparative Group (n = 133)
**N %**	**N %**
Age in years, median (IQR)		50 (40–56)	49 (39–55)
Sex	Male	74 (55.64)	74 (55.64)
	Female	59 (44.36)	59 (44.36)
Residence	Urban	124 (93.23)	121 (90.98)
	Rural	9 (6.77)	12 (9.02)
Educational status	Unable to read and write	12 (9.02)	22 (16.54)
	Can read and write	55 (41.35)	43 (32.33)
	Primary school	17 (12.78)	14 (10.53)
	Secondary school	17 (12.78)	11 (8.27)
	Diploma and above	32 (24.06)	43 (32.33)
Occupational status	Student	0 (0)	2 (1.5)
	Unemployed	35 (26.32)	26 (19.55)
	Government employee	15 (11.28)	16 (12.03)
	Private employee	30 (22.56)	48 (36.09)
	Farmer	0 (0)	2 (1.5)
	House wife	45 (33.83)	26 (19.55)
	Others ^**ab**^	8 (6.02)	13 (9.77)

Others ^**ab**^: Businessman & Daily labor

### Anthropometric, blood pressure and clinical characteristics

In the current study, the mean HC of the T2DM patients was significantly higher as compared with comparative group (96.28 ± 14.64 kg vs 92.86 ± 13.39 kg) with a statistically significant difference between the two groups (*p* = 0.05). Additionally, the median of WC, SBP, and FBG of T2DM patients were significantly higher as compared with comparative group (93 cm vs 87 cm), (134 mmHg vs 126 mmHg), and (183 mg/dl vs 83 mg/dl) with a statistically significant difference between the two groups (*p* = 0.01, 0.002, and 0.001), respectively Table 2.

**Table 2:** Anthropometric, blood pressure and clinical characteristics of adult patients with type 2 diabetes mellitus and a comparative group at selected hospitals in Garowe, Puntland, Somalia, from October 1 to November 15, 2024 (n=266).

Variable	T2DM Patients (n = 133)	Comparative Group (n = 133)	*p*-value
Weight (kg), mean ± SD	74.63 ± 12.94	72.62 ± 11.85	0.18
Height (m), median (IQR)	1.71(1.65-1.76)	1.70 (1.65-1.78)	0.89
BMI (kg/m ^2^), median (IQR)	25.6 (23.4-28.3)	24.5 (22.8-27.8)	0.11
WC (cm), median (IQR)	93 (82-104)	87 (78-99)	0.01*
HC (cm), mean ± SD	96.28 ± 14.64	92.86 ± 13.39	0.05*
WHR, median (IQR)	0.96 (0.93-0.99)	0.96 (0.91-0.98)	0.20
SBP (mmHg), median (IQR)	134 (123-148)	126 (120-136)	0.002*
DBP (mmHg), median (IQR)	78 (70-88)	75 (69-82)	0.14
FBG level (mg/dl), median (IQR)	183 (138-237)	83 (75-91)	<0.001*
HbA1C (%), median (IQR)	8.85 (6.95- 10.7)	**_________**	
Duration DM illness (years), median (IQR)	4 (2-8)	—	

**Note:** * *p*-value <0.05, statistically significant

### Comparison of hematological parameters of the study participants

In the present study, the median values of absolute neutrophil count (3.13x10^6^ cells/L vs 2.67x10^6^ cells/L), MCHC (29.6 g/dl vs 29.3 g/dl), and PDW (15.9 fL vs 15.7 fL) were significantly higher in T2DM patients compared to the comparative group, with statistically significant differences between the two groups *(p* = 0.01, 0.02, and <0.001, respectively). Conversely, the median value of MCV (93.4 fL vs 95.4 fL), and the mean value of MPV (11.35 fL vs. 11.84 fL) were lower in T2DM patients compared to the comparative group, with statistically significant differences between the two groups (*p* = 0.005, and 0.003, respectively) Table 3.

**Table 3:** Comparison of hematological parameters between adult patients with type 2 diabetes mellitus and a comparative group at selected hospitals in Garowe, Puntland, Somalia, from October 1 to November 15, 2024 (n =  266).

Variable	T2DM Patients (n = 133)	Comparative groups (n = 133)	*P*-value
WBC count (10^9^cells/L), median (IQR)	6.48 (5.54-8.11)	6.09 (5.18-7.5)	0.06
Neutrophil (10^9^cells/L), median (IQR)	3.13 (2.46-4.31)	2.67 (2.15-3.78)	0.01*
Lymphocyte (10^9^cells/L), median (IQR)	2.25 (1.71-2.76)	2.34 (1.89-2.76)	0.57
Monocyte (10^9^cells/L), median (IQR)	0.57 (0.38-0.94)	0.64 (0.45-1.04)	0.19
Eosinophil (10^9^cells/L), median (IQR)	0.17 (0.11-0.28)	0.18 (0.12-0.29)	0.56
Basophil (10^9^cells/L), median (IQR)	0.01 (0.01-0.02)	0.01 (0.01-0.02)	0.27
RBC count (10^12^cells/L), median (IQR)	4.78 (4.43-5.14)	4.68 (4.37-5.08)	0.53
Hgb (g/dl), median (IQR)	13.1 (12.1-14.0)	13.1 (11.9-14.2)	0.80
HCT (%), median (IQR)	43.9 (40.2-47.6)	44.6 (40.3-48.4)	0.28
MCV (fL), median (IQR)	93.4 (88.6-96.4)	95.4 (91.0-98.2)	0.005*
MCH (Pg), median (IQR)	27.5 (26.6-28.7)	28.1 (27.0-28.8)	0.18
MCHC (g/dl), median (IQR)	29.6 (28.7-30.4)	29.3 (28.7-29.9)	0.02*
RDW-CV (%), median (IQR)	13.4 (12.8-14.4)	13.3 (12.8-14.1)	0.48
RDW-SD (fL), median (IQR)	43.4 (41.0-46.9)	44.5 (43.1-47.1)	0.06
Platelet count (10^9^cells/L), median (IQR)	247 (202-300)	242 (202-283)	0.53
MPV (fL), mean ± SD	11.35 ± 1.51	11.84 ± 1.15	0.003*
PDW (fL), median (IQR)	15.9 (15.7-16.2)	15.7 (15.5-15.9)	<0.001*
PCT (%), median (IQR)	0.276 (0.237-0.326)	0.281 (0.239-0.334)	0.57

**Note**: * *P*-value <0.05, statistically significant.

### Prevalence of hematological abnormalities of the study participants

In the current study, the most prevalent of hematological abnormalities among T2DM patients were anemia (31.57%; 95% CI: 23.7–40.2%), monocytosis (23.3%; 95% CI: 16.4–31.4%), thrombocytopenia (7.5%; 95% CI: 3.6–13.3%), and leukocytosis (6.8%; 95% CI: 3.1–12.5%). Among the comparative group, the most common abnormalities were anemia (30.8%; 95% CI: 23.1–39.4%), monocytosis (27.8%; 95% CI: 20.4–36.2%), and thrombocytopenia (6.8%; 95% CI: 3.1–12.5%) Table 4.

**Table 4:** Prevalence of hematological abnormalities between adult patients with type 2 diabetes mellitus and a comparative group at selected hospitals in Garowe, Puntland, Somalia, from October 1 to November 15, 2024 (n =  266).

Hematological abnormalities	T2DM (95% CI) N = 133	Comparative (95% CI) N = 133	Cutoff value
Anemia	31.57% (23.7-40.2%)	30.8% (23.1- 39.4%)	<12 female, < 13 male g/dl [25]
Leukocytosis	6.8% (3.1-12.5%)	4.5% (1.6-9.5%)	>11.0 × 10^9^/L [26]
Neutrophilia	3% (0.8-7.5%)	3% (0.8-7.5%)	>8.0 × 10^9^/L [26]
Neutropenia	4.5% (1.6-9.5%)	4.5% (1.6-9.5%)	<1.5 × 10^9^/L [26]
Lymphocytosis	3% (0.8-7.5%)	0.7% (0.01-4.0)	> 4.0 × 10^9^/L [26]
Lymphopenia	2.25% (0.4-6.4%)	0.7% (0.01-4.0%)	<1.0 × 10^9^/L [26]
Monocytosis	23.3% (16.4-31.4)	27.8% (20.4-36.2%)	>1.0 × 10^9^/L [26]
Monocytopenia	3% (0.8-7.5%)	0.7% (0.01-4.0%)	<0.2 × 10^9^/L [26]
Thrombocytosis	3.76% (1.2-8.5%)	3% (0.8-7.5%)	>450 × 10^9^/L [27]
Thrombocytopenia	7.5% (3.6-13.3%)	6.8 (3.1-12.5%)	<150 × 10^9^/L [28]

### Associated factors of hematological abnormalities among adult patients with type 2 diabetes mellitus

This study utilized bivariable and multivariable logistic regression models to identify factors associated with hematological abnormalities among T2DM patients. Based on the bivariable analysis, age, sex, occupational status, smoking, oral hypoglycemic therapy used, and glycemic control were significantly associated with anemia in the COR (*p* < 0.25). However, in multivariable logistic regression model, poor glycemic control was significantly associated with anemia (*p* < 0.05). The odds of developing anemia with poor glycemic control of T2DM patients were 3.06 times more likely (AOR = 3.06, 95% CI: 1.06–8.8) compared to those with good glycemic control Table 5.

**Table 5:** Associated factors of anemia among adult patients with type 2 diabetes mellitus at selected hospitals in Garowe, Puntland, Somalia, from October 1 to November 15, 2024 (n = 133).

Variables	Categories	Anemia		COR (95% CI)	P-Value	AOR (95% CI)	P-Value
**Yes (%)**	**No (%)**
**Age in years**	**18-37**	6 (20.0)	24 (80.0)	0.78 (0.22-2.69)	0.70	0.76 (0.20-2.86)	0.68
	**38-57**	29 (39.2)	45 (60.8)	2.02 (0.76-5.34)	0.15*	2.63 (0.88-7.85)	0.08
	**58-65**	7 (24.1)	22 (75.9)	1.0			
**Sex**	**Male**	27 (36.5)	47 (63.5)	1.68 (0.79-3.57)	0.18*	1.53 (0.34-6.73)	0.57
	**Female**	15 (25.4)	44 (74.6)	1.0			
**Occupational status**	**Unemployed**	14 (40.0)	21 (60.0)	1.0			
	**Private/Government employed**	12 (26.7)	33 (73.3)	0.54 (0.21-1.40)	0.21*	0.42 (0.14-1.27)	0.13
	**House wife**	11 (24.4)	34 (75.6)	0.48 (0.18-1.26)	0.14*	0.55 (0.12-2.44)	0.44
	**Others** ^**ab**^	5 (62.5)	3 (37.5)	2.5 (0.51-12.2)	0.26	1.35 (0.22-8.21)	0.74
**Smoking**	**Non-Smoker**	33 (29.2)	80 (70.8)	1.0			
	**Smoker**	9 (45.0)	11 (55.0)	1.98 (0.75-5.23)	0.17*	1.30 (0.38-4.39)	0.67
**Oral hypoglycemic therapy used**	**Glibenclamide**	4 (22.2)	14 (77.8)	1.0			
	**Metformin**	27 (29.7)	64 (70.3)	1.47 (0.45-4.89)	0.52	2.12 (0.56-7.91)	0.26
	**Metformin / Glipizide**	5 (41.7)	7 (58.3)	2.5 (0.50-12.4)	0.26	1.47 (0.26-8.32)	0.66
	**Others** ^**cd**^	6 (50.0)	6 (50.0)	3.5 (0.71-17.1)	0.12*	4.72 (0.77-28.7)	0.09
**Glycemic control**	**Good control**	6 (16.7)	30 (83.3)	1.0			
	**Poor control**	36 (37.1)	61 (62.9)	2.95 (1.12-7.77)	0.03*	3.06 (1.06-8.8)	0.04*

**Note**: COR: Crud Odds Ratio, AOR: Adjusted Odds Ratio, CI: Confidence Interval, *: Statistically significant association, others ^**ab**^: Businessman & Daily wadge labor; others ^**cd**^: Combination of Metformin / Glibenclamide and non-oral hypoglycemic therapy

Additionally, in the bivariable analysis, occupational status, BMI, SBP, and oral hypoglycemic therapy used showed significantly associated with leukocytosis in the COR (*p* < 0.25). However, in multivariable logistic regression analysis model, SBP > 140 level was remained associated with leukocytosis (*p* < 0.05). The odds of developing leukocytosis with SBP > 140 level of T2DM patients were 6.09 times more likely (AOR = 6.09, 95% CI: 1.09–33.9) compared to those with an SBP ≤ 140 Table 6.

**Table 6:** Associated factors of leukocytosis among adult patients with type 2 diabetes mellitus at selected hospitals in Garowe, Puntland, Somalia, from October 1 to November 15, 2024 (n=133).

Variables	Categories	Leukocytosis		COR (95% CI)	P-Value	AOR (95% CI)	P-Value
**Yes (%)**	**No (%)**
**Occupational status**	**Unemployed**	5 (14.3)	30 (85.7)	1.0			
	**Private/Government employed**	1 (2.2)	44 (97.8)	0.13 (0.01-1.22)	0.08 *	0.12 (0.01-1.23)	0.07
	**House wife**	2 (4.4)	43 (95.6)	0.27 (0.05-1.53)	0.14 *	0.19 (0.02-1.36)	0.10
	**Others** ^**ab**^	1 (12.5)	7 (87.5)	0.85 (0.08-8.54)	0.89	0.62 (0.03-10.6)	0.74
**BMI**	**Underweight**	2 (28.6)	5 (71.4)	7.2 (1.05-49.3)	0.04 *	5.30 (0.51-55.1)	0.16
	**Normal weight**	3 (6.0)	47 (94.0)	1.14 (0.25-5.36)	0.86	1.60 (0.26-9.67)	0.60
	**Over weight**	4 (5.3)	72 (94.7)	1.0			
**SBP (mmHg)**	**≤140**	3 (3.6)	81 (96.4)	1.0			
	**>140**	6 (12.2)	43 (87.8)	3.76 (0.89-15.8)	0.07 *	6.09 (1.09-33.9)	0.04*
**Oral hypoglycemic therapy used**	**Glibenclamide**	1 (5.6)	17 (94.4)	1.0			
	**Metformin**	4 (4.4)	87 (95.6)	0.78 (0.08-7.43)	0.83	0.86 (0.07-9.57)	0.91
	**Metformin / Glipizide**	1 (8.3)	11 (91.7)	1.5 (0.08-27.4)	0.77	1.77 (0.08-37.7)	0.71
	**Others** ^**cd**^	3 (25.0)	9 (75.0)	5.7 (0.51-62.7)	0.16 *	5.97 (0.38-93.4)	0.20

**Note**: COR: Crud Odds Ratio, AOR: Adjusted Odds Ratio, CI: Confidence Interval, *: Statistically significant association, others ^**ab**^: Businessman & Daily wadge labor; others ^**cd**^: Combination of Metformin / Glibenclamide and non-oral hypoglycemic therapy

On other hands, in the bivariable analysis, age, occupational status, SBP, and physical activity showed a significant association with monocytosis in the COR (*p* < 0.25). However, following multivariable analysis all of them were not significantly associated with monocytosis (*p* > 0.05).

### Correlation of hematological parameters with anthropometric, blood pressure and clinical variables among adult patients with type 2 diabetes mellitus

Pearson and Spearman’s correlation tests were applied to assess the correlation between BMI, WHR, DBP, SBP, duration of DM illness, FBG and hematological parameters among T2DM patients. The analysis revealed that there was a weak positive correlation between MPV and BMI (rho = 0.21, *p* = 0.01). In the RDW-SD (rho = 0.19, *p* = 0.02) showed a significant but very weak positive correlation with SBP, while RDW-CV (rho = 0.24, *p* = 0.004) displayed a significant weak positive correlation with SBP. Additionally, RDW-CV (rho = 0.19, *p* = 0.02) demonstrated a significant but very weak positive correlation with DBP.

Regarding the duration of DM illness, PCT (rho = −0.17, *p* = 0.04) showed a significant but very weak negative correlation. Additionally, RBC (rho = −0.20, *p* = 0.02) exhibited a significant weak negative correlation, whereas MCV (rho = 0.22, *p* = 0.01), and MCH (rho = 0.27, *p* = 0.001) demonstrated significant weak positive correlations.

Additionally, FBG exhibited a significant but very weak negative correlation with the absolute monocyte count (rho = −0.18, *p* = 0.03). Meanwhile, MCV (rho = −0.20, *p* = 0.01), RDW-CV (rho = −0.21, *p* = 0.01), and RDW-SD (rho = −0.29, *p* = 0.001) showed significant weak negative correlations. In contrast, RBC (rho = 0.17, *p* = 0.04) demonstrated a significant but very weak positive correlation with FBG, while PDW (rho = 0.20, *p* = 0.02) exhibited a significant weak positive correlation with FBG. In the present study, a statistically significant correlation was not observed between hematological parameters and WHR Table 7.

**Table 7:** Correlation of hematological parameters with anthropometric, blood pressure, and clinical variables among adult type 2 diabetes mellitus patients at selected hospitals in Garowe, Puntland, Somalia, from October 1 to November 15, 2024 (n=133).

Variable	BMI	WHR	SBP	DBP	DM duration	FBG
**Rho (*p*)**	**Rho (*p*)**	**Rho (*p*)**	**Rho (*p*)**	**Rho (*p*)**	**Rho (*p*)**
WBC	−0.07 (0.45)	−0.03 (0.70)	0.13 (0.13)	0.12 (0.14)	0.03 (0.70)	0.05 (0.51)
Neutrophil	−0.004 (0.96)	−0.06 (0.45)	0.12 (0.15)	0.11(0.19)	−0.04 (0.64)	0.07 (0.36)
Lymphocyte	−0.04 (0.63)	−0.03 (0.70)	−0.11 (0.17)	0.01 (0.88)	−0.07 (0.41)	0.02 (0.81)
Monocyte	−0.08 (0.32)	−0.02 (0.78)	0.09 (0.28)	0.001 (0.98)	0.09 (0.26)	−0.18 (0.03) *
Eosinophil	0.002 (0.98)	0.08 (0.33)	0.04 (0.64)	0.002 (0.97)	0.04 (0.64)	0.12 (0.16)
Basophil	−0.09 (0.27)	−0.12 (0.16)	0.10 (0.23)	−0.10 (0.21)	−0.05 (0.53)	−0.04 (0.62)
RBC	−0.02 (0.78)	−0.003(0.96)	−0.14 (0.10)	−0.08 (0.34)	−0.20 (0.02) *	0.17 (0.04) *
Hgb	−0.04 (0.65)	0.05 (0.55)	−0.15 (0.07)	−0.04 (0.62)	−0.02 (0.81)	0.12 (0.14)
Hct	−0.02 (0.80)	−0.008 (0.92)	−0.09 (0.27)	−0.07 (0.41)	−0.04 (0.64)	0.07 (0.39)
MCV	0.02 (0.78)	−0.03 (0.65)	0.01 (0.87)	−0.07 (0.38)	0.22 (0.01) *	−0.20 (0.01) *
MCH	−0.04 (0.67)	0.11 (0.17)	−0.11 (0.20)	−0.04 (0.63)	0.27 (0.001) *	−0.11 (0.20)
MCHC	−0.15 (0.08)	0.07 (0.41)	−0.08 (0.33)	−0.03 (0.72)	0.09 (0.26)	0.07 (0.38)
RDW-CV	0.04 (0.59)	0.01 (0.91)	0.24 (0.004) *	0.19 (0.02) *	−0.01 (0.88)	−0.21 (0.01) *
RDW-SD	0.04 (0.64)	0.02 (0.81)	0.19 (0.02) *	0.09 (0.28)	0.15 (0.06)	−0.29 (0.001) *
PLT	−0.06 (0.51)	−0.05 (0.56)	−0.02 (0.82)	0.05 (0.51)	−0.14 (0.09)	0.03 (0.69)
MPV	0.21 (0.01) *	−0.04 (0.68)	−0.10 (0.27)	−0.16 (0.06)	−0.10 (0.26)	−0.11 (0.20)
PDW	−0.04 (0.63)	−0.07 (0.40)	−0.02 (0.80)	0.01 (0.93)	−0.04 (0.58)	0.20 (0.02) *
PCT	0.02 (0.78)	−0.04 (0.59)	−0.10 (0.21)	−0.04 (0.58)	−0.17 (0.04) *	0.01 (0.87)

**Note:** * Correlation is significant at the 0.05 level; *p*-value <0.05 is considered as statistically significant; rho is spearman’s correlation coefficient; p is p-value.

## Discussion

The current study reveals that the mean (SD) and median (IQR) values of absolute neutrophil count, MCV, MCHC, MPV, and PDW are statistically significantly different between T2DM patients and the comparative group (*p* < 0.05). Regarding MCV and MCHC, the present study shows that they are statistically significantly different between T2DM patients and the comparative group. This result is consistent with findings reported in Nigeria [29], and Dessie, Ethiopia [30]. This could be explained by several risk factors, including hyperglycemia, hyperosmolarity, OS, inflammation and lipid metabolic disorders, which may impact RBC metabolism in diabetic patients. These factors contribute to increased aggregation, reduced cell deformability, and decreased membrane fluidity. Consequently, these changes lead to a reduced survival rate, altered morphology, size, and functions of RBCs [8].

In the present study, the prevalence of anemia among T2DM patients is 31.57% (95% CI: 23.3–40.2%). This is in line with the study done in Australia (24%) [31], Brazil (34.2%) [32], Iran (30.4%) [33], and Harar, Ethiopia (34.8%) [34]. However, this finding is relatively higher than a study conducted in Debre Berhan, Ethiopia (20.1%) [35], and China (22%) [36]. Conversely, this prevalence is comparatively lower than study conducted in, India (67%) [17], Nigeria (45.2%) [37], and Cameroon (41.4%) [38]. These discrepancies could be attributed to variations in sample size, lifestyle, altitude, feeding habits, duration of DM illness, sociodemographic, economic status, and access to healthcare. Notably, anemia was also observed among 30.8% of the comparative group. The comparable prevalence of anemia between T2DM patients and apparently healthy controls suggests that anemia may represent a broader public health concern within the study population rather than a condition exclusively attributable to T2DM. This finding indicates that additional factors, including nutritional deficiencies, infectious diseases, socioeconomic conditions, and other unmeasured population-level determinants, may contribute to the burden of anemia in the region. Therefore, while diabetes may be associated with hematological alterations, the high prevalence of anemia observed in both groups underscores the need to consider anemia within a wider population-health context.

A logistic regression analysis of the current study shows that patients who had poor glycemic control were more likely to have anemia compared to those with good glycemic control. This finding is consistent with studies reported in Pakistan [39], Jordan [40], India [41], and Ethiopia [35]. However, given the cross-sectional nature of the study, this association should not be interpreted as evidence of causality. Rather, poor glycemic control may contribute to or exacerbate hematological abnormalities through mechanisms involving oxidative stress, chronic inflammation, impaired erythropoietin regulation, and altered erythrocyte survival [42]. Considering the substantial prevalence of anemia observed among the comparative group, poor glycemic control is more appropriately viewed as a contributing factor that may worsen existing hematological disturbances rather than the sole underlying cause of anemia.

With respect to WBC indices, patients with T2DM exhibited a significantly higher absolute neutrophil count compared with the comparative group, consistent with findings from Saudi Arabia [43], Nigeria [29], Sudan [44], and Debre Berhan, Ethiopia [22]. Furthermore, WBC abnormalities including monocytosis, leukocytosis, neutrophilia, and lymphocytosis were observed among T2DM patients, with prevalence rates comparable to those reported in Debre Berhan, Ethiopia [22]. The reason for these variations might be that the elevated WBC count in the T2DM patients is in aligns with the increased oxidative stress triggered by the high levels of hyperglycemia. However, polymorphonuclear and mononuclear WBCs can be activated by AGEs, angiotensin II, and cytokines in a state of hyperglycemia [45]. Leukocytosis is a classical marker of inflammation and evidence from epidemiological studies suggests an association between WBC count, a non-specific marker of inflammation, and diabetes risk [20]. A persistent, low-grade inflammatory condition results from defects in the way that insulin acts on the primary insulin-sensitive tissues. The release of proinflammatory cytokines, which encourage leukocyte differentiation and maturation, can be triggered by extravascular stimuli as well as intra-arterial inflammation [46].

Among T2DM patients in the current study, 123 (92.5%) had normal neutrophil counts, whereas 6 [4.5% (95% CI: 1.6%−9.5%)] were found to have neutropenia. Similarly, a study conducted in Debre Berhan, Ethiopia, reported 3.7% neutropenia. Diabetic neutrophils have been associated with impaired deformability, chemotaxis, phagocytosis, bactericidal activity, and they also die sooner than normal [47].

Logistic regression analysis shows that the leukocytosis among T2DM patients with an SBP > 140 is more likely compared to those with an SBP ≤ 140. This variation might be due to AGEs produced in diabetes quench nitric oxide in vitro and could decrease nitric oxide’s vasodilatory action. Additionally, it activated nuclear factor-kappa B (NF-κB), which caused endothelial cells to produce vasoconstrictor endothelin-1. Consequently, endothelin-1 raises SBP since it is a strong vasoconstrictor [48].

Regarding platelet indices, the present study reveals that MPV is lower in the T2DM patients compared to the comparative groups. Comparable findings were reported in studies conducted in Saudi Arabia [43], and Sudan [44], while, contrary to a studies conducted in India [49], and Dessie, Ethiopia [30]. This variation might be due to the effect of oxidative stress. Increased reactive oxygen species in diabetes causes nonenzymatic glycation of platelet surface proteins, which causes an excess of AGEs to accumulate. Some of these AGEs cause externalization of platelet membrane phosphatidylserine that may cause changes in protein structure and membrane lipid dynamics [50]. Additionally, increased platelet levels can accelerate atherosclerosis and are linked to both macrovascular and microvascular issues. They may be elevated in conditions such as peripheral arterial disease, coronary artery disease and myocardial infarction [51].

Additionally, the current study indicates that the PDW is higher in the T2DM patients compared to the comparative groups. This results is similar to the findings reported in Sudan [44], and Disse, Ethiopia [30]. This difference may be due to non-enzymatic glycation of platelet surface proteins and activation of protein kinase C, which reduces platelet membrane fluidity and increases activation. The size difference between activated and inactivated platelets in T2DM patients leads to pseudopodia formation, altering platelet morphology from biconcave discs to spheres, ultimately affecting PDW. [52].

Thrombocytopenia was observed in 7.5% (95% CI: 3.6–13.3%) of T2DM patients, while 3.75% (95% CI: 1.2–8.5%) had thrombocytosis. These findings are comparable to a study conducted in Gondar, Ethiopia, which reported a thrombocytopenia prevalence of 5.3%, although the proportion of thrombocytosis in that study (1.1%) was notably lower than the value observed in the current study [53]. The possible justification might be that platelets in diabetic patients have dysregulated signaling, increasing activation and aggregation, which are linked to endothelial dysfunction, poor clot breakdown, and increased clotting, essential for hemostasis [54]. Elevated platelet reactivity in diabetic patients is linked to hyperglycemia, insulin deficiency and resistance, metabolic conditions like obesity and dyslipidemia, increased inflammation, and other cellular abnormalities. [15].

The present study assessed the correlation between hematological parameters and anthropometric indices, blood pressure, FBG, and duration of illness among patients with T2DM. In this study, FBG demonstrated significant correlations with several hematological parameters. Specifically, absolute monocyte count, MCV, RDW-CV, and RDW-SD showed weak negative correlations with FBG, whereas RBC count and PDW exhibited weak positive correlations. These findings are in line with a study conducted in Dessie, Ethiopia [30]. This could be a result of inadequate insulin production, poor glycemic control, or insulin intolerance in diabetic individuals [29].

Regarding blood pressure parameters, SBP showed a weak positive correlation with RDW-SD and RDW-CV, while RDW-CV was also weak positive correlation with DBP. These findings are consistent with previous reports from Dessie, Ethiopia [30]. The relationship between RDW and blood pressure may be explained by underlying inflammatory mechanisms and activation of the renin–angiotensin–aldosterone system (RAAS). Increased angiotensin II activity can stimulate erythropoietin production, leading to the release of immature erythrocytes into circulation, thereby increasing RDW. Furthermore, inflammation-related alterations in iron metabolism and bone marrow activity may contribute to anisocytosis. Elevated RDW has also been linked to endothelial dysfunction, impaired microcirculation, and tissue hypoxia, all of which are associated with hypertension [55].

With respect to the duration of DM illness, RBC count and PCT demonstrated weak negative correlations, whereas MCV and MCH showed weak positive correlations with the duration of T2DM. These findings are comparable with those reported in Debre Berhan, Ethiopia. A possible explanation for this variation is that diabetics with chronic complaints and vascular complications consistently exhibit long-term hyperglycemia, which results in a decreased RBC count and an elevated RDW [22].

Additionally, MPV showed a weak positive correlation with BMI, which is consistent with findings from Debre Berhan, Ethiopia [22], but contrasts with studies conducted in India [56] that reported no such association. This discrepancy may be due to differences in study populations or sample size. The observed relationship between MPV and BMI could be explained by obesity associated with systemic inflammation, and certain inflammatory conditions contribute to platelet activation, which in turn causes the formation of larger platelets [57]. The strong correlation between BMI and hypertension suggests that changes in cytokine levels and platelet counts are closely linked to fat distribution, a key indicator of inflammation [15].

Some limitations of our study were that we did not assess the levels of iron, vitamin B12, folate, renal function, lipid profile, inflammatory markers and cardiovascular disease burden were not quantified and a standardized comorbidity index were not applied.

## Conclusion and recommendations

In the current study, the median values of absolute neutrophil count, MCHC, and PDW were significantly higher, while the mean and median values of MCV, and MPV were significantly lower in T2DM patients compared with the comparative group. Anemia, monocytosis, and thrombocytopenia, were the most prevalent hematological abnormalities in both T2DM patients and the comparative group. Poor glycemic control was significantly associated with anemia among T2DM patients, while SBP > 140 mmHg was significantly associated with leukocytosis. Furthermore, BMI, SBP, DBP, FBG and duration of diabetes demonstrated both positive and negative correlations that were statistically significant with some hematological parameters among T2DM patients. We recommend future longitudinal studies with larger sample size with assessment of renal function, lipid profile, inflammatory markers, cardiovascular disease burden and standardized comorbidity index to demonstrate a causal relationship between hematological abnormalities and T2DM.

**Abbreviations:** AGE, Advanced Glycation End Products; AOR, Adjusted Odds Ratio; BMI, Body Mass Index; BP, Blood Pressure; CI, Confidence Interval; COR, Crude Odds Ratio; DBP, Diastolic Blood Pressure; DM, Diabetes Mellitus; FBG, Fasting Blood Glucose; HCT, Hematocrit; Hgb, Hemoglobin; IQR, Interquartile Range; K2-EDTA, Dipotassium Ethylenediaminetetraacetic Acid; MCH, Mean Corpuscular Hemoglobin; MCHC, Mean Corpuscular Hemoglobin Concentration; MCV, Mean Corpuscular Volume; MPV, Mean Platelet Volume; PCT, Plateletcrit; PDW, Platelet Distribution Width; PLT, Platelets; RBC, Red Blood Cell; RDW-CV, Red Cell Distribution Width–Coefficient of Variation; RDW-SD, Red Cell Distribution Width–Standard Deviation; SBP, Systolic Blood Pressure; T2DM, Type 2 Diabetes Mellitus; WBC, White Blood Cell; WHO, World Health Organization; WHR, Waist-to-Hip Ratio.

## Supporting information

S1 File Inclusivity in global research questionnaire. (DOCX)

S2 File Questionnaire. (DOCX)

S3 File Hematological parameters of T2DM and Comparative group raw data. (XLSX)
