# Supplementary material for: Hematological parameters and associated factors among adult patients with type 2 diabetes mellitus attending selected hospitals in Garowe, Puntland, Somalia: A comparative cross-sectional study
Source: PLoS One. 2026 Jul 16;21(7):e0353173. doi: 10.1371/journal.pone.0353173 (PMC13375008; doi:10.1371/journal.pone.0353173)
Supplement: S1 File — (DOCX) [file pone.0353173.s001.docx]

Inclusivity in global research

PLOS’ policy on inclusivity in global research aims to improve transparency in the reporting of research performed outside of researchers’ own country or community and ensures that PLOS publications reporting global research adhere to high standards for research ethics and authorship. Authors of relevant research articles may be asked to complete the questionnaire below, which outlines ethical, cultural, and scientific considerations specific to inclusivity in global research. This questionnaire may be requested when researchers have travelled to a different country to conduct research, if research uses samples collected in another country, research with Indigenous populations or their lands, or if research is on cultural artefacts. Researchers travelling to another country solely to use laboratory equipment will not normally be required to complete the questionnaire. However, the questionnaire can be requested at the journal’s discretion for any submission – if you have been requested to complete this questionnaire by the PLOS journal you submitted to, please do so.

Please complete the questionnaire below and include this as a Supporting Information file with your manuscript. Note that if your paper is accepted for publication, this checklist will be published with your article in the supporting information files. Please ensure that you reference the checklist in the main body of your manuscript. We suggest adding a subsection ‘Inclusivity in global research’ to your Methods section and adding the following sentence: “Additional information regarding the ethical, cultural, and scientific considerations specific to inclusivity in global research is included in the Supporting Information (SX Checklist)”

The questions have been designed to be applicable to a wide range of study types, and there are subsections for both human subjects research and non-human subjects research. If any of the questions are not relevant to your research please mark them as “N/A” as appropriate.

**Ethical considerations, permits and authorship**

*This section is applicable to all research types.*

Provide details as to who granted permissions and/or consent for the study to take place in the Methods section of your manuscript. This should include the names of **all** ethics boards, governmental organizations, community leaders or other bodies that provided approval for the study. If individuals provided approval refer to these people by their role or title but do not list their name(s).

Reported on page number: Six and Seven

If there were any deviations from the study protocol after approval was obtained please provide details of these changes in the Methods section of your manuscript.
Did this study involve local collaborators that are residents of the country where the research was conducted or members of the community studied? If you do not have any authors from said communities, please provide an explanation for this below.

Reported on page number: Not Applicable (NA)

**Response:**

This hospital-based comparative cross-sectional study was conducted from October 1 to November 15, 2024, at Garowe General Hospital, Arafat International Hospital, and Dalab Hospital in Garowe, Puntland, Somalia. Garowe, the capital and administrative centre of Puntland State in the Nugaal region of northeastern Somalia, has experienced substantial urban growth since Puntland's establishment in 1998 and serves as a key political and economic hub. The selected hospitals provide comprehensive healthcare services, including emergency care, chronic disease management, antiretroviral therapy, surgery, paediatrics, gynaecology, obstetrics, and dental care for both inpatient and outpatient populations.

The principal researcher was an M.Sc. international student and a citizen of Somalia, enrolled at the College of Health and Medical Sciences, Haramaya University, Ethiopia, an institution in Ethiopia and conducted experimental fieldwork in Somalia. Although the principal researcher is a resident of Somalia, the study was carried out with the support and cooperation of local hospital administrations and healthcare professionals at the participating institutions, who facilitated participant recruitment and sample collection.

There were no formal academic collaborators or co-authors from Somali institutions or the specific communities studied. This was primarily due to constraints in establishing formal research partnerships within the timeframe and scope of the M.Sc. project. We acknowledge that the absence of formal local academic collaboration is a limitation. Efforts were made to ensure ethical and culturally appropriate conduct by engaging with hospital authorities, adhering to local regulations, and respecting community norms. Future research will aim to build stronger collaboration with local researchers and institutions to enhance contextual relevance and inclusivity.

Everyone listed as an author should meet PLOS’ criteria for authorship and all individuals who meet these criteria should be included in the author byline, rather than the acknowledgements. For further information please see the journal’s Authorship Policy.

**Human subjects research (e.g. health research, medical research, cross-cultural psychology)**

Did you obtain written informed consent from a representative of the local community or region before the research took place? How did you establish who speaks for the community? Details of written informed consent obtained from study participants should be reported separately in the Methods section of your manuscript.

**Response:**

A letter of permission was obtained from the district administrative health bureau. obtained before the initiation of the study. Authorization to conduct the research was obtained from the administrative and clinical leadership of the participating hospitals (Garowe General Hospital, Arafat International Hospital, and Dalab Hospital), who act as institutional gatekeepers for research conducted within their facilities.

These hospital authorities were identified as appropriate representatives based on their official roles in overseeing clinical services, patient welfare, and research activities within their respective institutions. Permission was granted following review of the study objectives, procedures, and ethical considerations.

The study was conducted in accordance with applicable ethical standards, including respect for local norms and institutional requirements. All individual study participants provided written informed consent before enrolment, as detailed separately in the Methods section of the manuscript.

We acknowledge that broader community-level engagement beyond institutional approval was limited, and future research will aim to incorporate more comprehensive community representation and consultation where feasible.

How did members of the local community provide input on the aims of the research investigation, its methodology, and its anticipated outcome(s)?

**Response:**

Members of the local community were not formally involved in providing input on the aims, methodology, or anticipated outcomes of this research. The study was designed as part of an M.Sc. project by the principal researcher, with the research objectives and methods developed in consultation with academic supervisors at the enrolling institution.

However, the study was implemented in close coordination with the administrative and clinical staff of the participating hospitals (Garowe General Hospital, Arafat International Hospital, and Dalab Hospital). These local healthcare professionals provided practical input on the feasibility of the study procedures, participant recruitment, and alignment with routine clinical workflows, which helped ensure that the methodology was appropriate for the local healthcare setting.

Although this input improved the contextual applicability of the study, it did not constitute formal community engagement in shaping the research aims or anticipated outcomes. We acknowledge this as a limitation. Future research will aim to involve community representatives and local stakeholders more actively in the design and planning phases to enhance relevance, inclusivity, and local ownership of the research.

When engaging with the local community, how did you ensure that the informed consent documents and other materials could be understood by local stakeholders?

**Response:**

To ensure that informed consent documents and study materials were understandable to local stakeholders, all questionnaires and consent forms were initially prepared in English, translated into the local language (Afan-Somali), and then back-translated into English to ensure accuracy and consistency.

In addition, data collectors—who were familiar with the local language and context—received two days of training on the study procedures, including how to clearly explain the purpose, risks, and benefits of the study to participants. During the consent process, participants were allowed to ask questions, and explanations were provided in Afan-Somali to ensure full comprehension before obtaining written informed consent.

The questionnaires were also pre-tested before the actual data collection to assess clarity, cultural appropriateness, and comprehensibility, and necessary adjustments were made accordingly. These steps helped ensure that study materials were linguistically accurate, culturally appropriate, and easily understood by participants and local stakeholders.

Will the findings of the research be made available in an understandable format to stakeholders in the community where the study was conducted (e.g. via a presentation, summary report, copies of publications, etc.)? Please provide details of how this will be achieved.

**Response:**

Yes, the findings of this research will be made available to relevant stakeholders in the community where the study was conducted. The results will be shared with the participating hospitals (Garowe General Hospital, Arafat International Hospital, and Dalab Hospital) through summary reports and, where feasible, brief presentations to hospital administrators and healthcare staff. These summaries will highlight key findings, clinical implications, and recommendations in a clear and accessible format.

In addition, copies of the final thesis and any resulting publications will be made available to the participating institutions. Efforts will be made to communicate key findings in a concise and understandable manner, potentially including summaries translated into Af-Somali to enhance accessibility for local stakeholders.

These dissemination activities aim to ensure that the results are accessible, useful for local healthcare practice, and contribute to improving the management of diabetes and related haematological conditions in the study setting.

**Non-human subjects research using specimens/ animals collected as part of the study, or those housed in archival collections. Examples include archaeology, paleontology, botany and zoology.**

Did the permission you obtained from a local authority to perform the study include an agreement on access to outputs and benefit sharing? This may include procedures to enable fair distribution of the benefits and resources arising from the research performed. Please include any details of Prior Informed Consent and Benefit Sharing Agreements obtained. These may be required by field-specific regulations, for example the Convention on Biological Diversity (CBD) and the associated Nagoya Protocol.

Not Applicable (NA)

If the material used in your study was imported, please A) provide the year it was imported and B) indicate whether permits were obtained to import/export the materials used, C) provide details of any permits obtained. If this information is not available, please indicate this.

**Response:**

No biological samples or study materials were imported into or exported from Somalia for this research. All data and blood samples were collected, processed, and analyzed within the participating hospitals (Garowe General Hospital, Arafat International Hospital, and Dalab Hospital) in Garowe, Puntland, Somalia, during the study period (October–November 2024).

Laboratory analyses, including complete blood count (CBC), HbA1c, and fasting blood glucose (FBG), were conducted on-site using available hospital laboratory facilities and equipment. Therefore:

A) Year of importation: Not applicable
B) Import/export permits: Not applicable
C) Details of permits: Not applicable

If you used archival specimens, please state how the material used in your study was acquired by the institute it is held in and provide details of any permits obtained for the original excavations/ sample collection. If this information is not available, please indicate this.

**Response:**

No archival specimens were used in this study. All biological samples were collected prospectively from study participants during the study period (October 1 to November 15, 2024) at the participating hospitals (Garowe General Hospital, Arafat International Hospital, and Dalab Hospital) in Garowe, Puntland, Somalia.

The samples were obtained directly from consenting participants specifically for this research and were processed and analyzed according to standard clinical and laboratory procedures within the respective hospital laboratories. Therefore, information regarding the acquisition of archival materials or permits for prior collection/excavation is not applicable.

How was the potential cultural significance of the materials collected in your study to local communities considered in your research design? Were Indigenous peoples and/or local researchers and institutions involved with archaeological excavations / collection of specimens? If so, please provide a description of their involvement.

**Response:**

The materials collected in this study consisted of routine clinical blood samples obtained from consenting adult participants for the purpose of analyzing hematological parameters in diabetes. These types of biological samples are not considered to have specific cultural or heritage significance within the local context, as they are commonly collected in standard medical care and laboratory investigations.

The research design prioritized ethical considerations, including respect for participants, voluntary participation, confidentiality, and adherence to local clinical and institutional guidelines. Permission to conduct the study was obtained from the administrative and clinical leadership of the participating hospitals (Garowe General Hospital, Arafat International Hospital, and Dalab Hospital), and the study procedures were aligned with routine healthcare practices in these settings.

This study did not involve archaeological excavations or the collection of culturally sensitive materials. While Indigenous populations or formal local research institutions were not directly involved as research collaborators or co-authors, local healthcare professionals and hospital staff played an important role in facilitating participant recruitment, sample collection, and implementation of the study within the clinical environment.

We acknowledge that broader involvement of local researchers and institutions would strengthen contextual relevance and inclusivity, and future studies will aim to establish more formal collaborations at the community and institutional levels.

If your manuscript includes photographs of human remains please indicate whether authors obtained permission from descendants or affiliated cultural communities to do so.

**Response:**

This manuscript does not include any photographs of human remains. Therefore, obtaining permission from descendants or affiliated cultural communities is not applicable.
