# Supplementary material for: Hematological parameters and associated factors among adult patients with type 2 diabetes mellitus attending selected hospitals in Garowe, Puntland, Somalia: A comparative cross-sectional study
Source: PLoS One. 2026 Jul 16;21(7):e0353173. doi: 10.1371/journal.pone.0353173 (PMC13375008; doi:10.1371/journal.pone.0353173)
Supplement: S2 File — (DOCX) [file pone.0353173.s002.docx]

## **Questionnaire**

**Questionnaire** **for T2DM patients**

Data collection questionnaire designed to conduct a study on Assessment of hematological parameters and associated factors among adult patients with type 2 diabetes mellitus attending hospitals in Garowe, Puntland, Somalia**:** A Comparative Cross-Sectional Study

**Identification code**_______________

| **S. No** | **Variable** | **Possible responses** | | |
| --- | --- | --- | --- | --- |
| **Part I.** | **Socio-demographic variables** |  | | |
| **101** | Age in years |  | | |
| **102** | Sex | 1. Male 2. Female | | |
| **103** | Residence | 1. Urban 2. Rural | | |
| **104** | Educational status | 1. Can’t read and write 2. Can read and write 3. Primary school 4. Secondary school 5. Diploma and above | | |
| **105** | Occupational status | 1. Student 2. Unemployed 3. Government employee 4. Private employee 5. Farmer 6. House wife 7. Other (specify)_____ | | |
| **Part II.** | **anthropometric and blood pressure variables** |  | | |
| **201** | Weight | ___________kg | | |
| **202** | Height | ____________m | | |
| **203** | Body mass index | ____________ kg / (m)^2^ | | |
| **204** | Waist Circumference | ____________cm | | |
| **205** | Hip Circumference | _____________cm | | |
| **206** | Waist to hip ratio |  | | |
| **207** | Blood pressure | SBP _________ mmHg  DBP ________ mmHg | | |
| **Part** **III** | **Life style variables** |  | | |
| **301** | Do you have a habit of smoking cigarette? | 1. Yes, I currently smoke 2. Yes, but I quit smoking 3. No, I have never smoked | | |
| **302** | Do you engage in physical activity? | 1. Yes 2. No | If 1 skip to Q303 | |
| **303** | If yes for Q302, how often do you activity? | - 1. Daily   2. 1-2 times a week   3. 3-5 times a week   4. Above 6 times a week | | |
| **304** | Do you consume milk? | 1. Yes 2. No | | If 1 skip to Q305 |
| **305** | If yes for Q304. How many liters per week do you drink? | 1. Less than 1 liter 2. One liter 3. Two to three liters 4. Above three liters | | |
| **Part IV** | **Clinical variables** |  | | |
| **401** | Current Oral hypoglycemic therapy used | 1. Glibenclamide 2. Metformin 3. Metformin + Glipizide 4. Others specify _________ | | |
| **402** | Duration of oral hypoglycemic therapy used | _____________ years | | |
| **403** | Duration of the DM illness | _____________ years | | |

| Data collector name _____________ Date ___________________ signature ____________ |
| --- |

**Questionnaire for comparative groups**

**Identification code**_______________

| **S. No** | **Variable** | **Possible responses** |
| --- | --- | --- |
| **Part I.** | **Socio-demographic variables** | |
| **101** | Age in years |  |
| **102** | Sex | - 1. Male 2. Female |
| **103** | Residence | - 1. Urban 2. Rural |
| **104** | Educational status | 1. Can’ read and write 2. Can read and write 3. Primary school 4. Secondary school 5. Diploma and above |
| **105** | Occupational status | - 1. Student   2. Unemployed   3. Government employee   4. Private employee   5. Farmer   6. House wife   7. Other (specify)_____ |
| **Part II.** | **Anthropometric and blood pressure variables** | |
| **201** | Weight | ___________kg |
| **202** | Height | ____________m |
| **203** | Body mass index | ____________ kg / (m)^2^ |
| **204** | Waist Circumference | ____________cm |
| **205** | Hip Circumference | _____________cm |
| **206** | Waist to hip ratio |  |
| **207** | Blood pressure | SBP _________ mmHg  DBP ________ mmHg |

| Data collector name ___________________ Date _______________ signature ___________ |
| --- |
